# Factors related to mammography adherence among women in Brazil: A scoping review

**DOI:** 10.1002/nop2.706

**Published:** 2020-11-24

**Authors:** Camila Brasil Moreira, V. Susan Dahinten, A. Fuchsia Howard, Ana Fátima Carvalho Fernandes, Janine Schirmer

**Affiliations:** ^1^ School of Nursing Federal University of São Paulo São Paulo Brazil; ^2^ School of Nursing University of British Columbia Vancouver BC Canada; ^3^ Department of Nursing Federal University of Ceará Fortaleza Brazil

**Keywords:** adherence, Brazil, breast cancer, mammography, scoping review, screening

## Abstract

**Aim:**

To explore and synthesize the literature on factors related to mammography screening adherence among women in Brazil.

**Design:**

A scoping review.

**Methods:**

We searched 11 databases for studies published between 2006–January 2020. All identified articles were screened, and data were extracted from eligible studies. We used the UK Government Social Research Service weight of evidence appraisal tool to appraise the quality of the included study.

**Results:**

From a total of 1,384 identified articles, 22 were retained. All included studies used quantitative, non‐experimental methods and all but two studies used cross‐sectional data. Quality of evidence varied across studies. We identified 41 factors that were investigated across the set of studies. Demographic and socio‐economic factors were the most commonly investigated, with older age, urban residence, living in the southeast of Brazil, higher level of education, higher income and private health insurance most consistently associated with mammography adherence.

## 1 INTRODUCTION

Worldwide, breast cancer is the most common malignant neoplasm among women, accounting for almost one in four cases of cancer and the greatest number of cancer‐related deaths in less developed countries (Bray et al., 2018). The incidence of breast cancer is rising in low‐ and middle‐income countries, as is the mortality rate, such that 62% of breast cancer deaths worldwide now occur in developing countries (Torre et al., 2017). The burden of breast cancer in Brazil, the largest country in South America, is similarly high. Breast cancer is the most prevalent cancer in Brazilian women, with prevalence rates ranging from 38.74/100,000 in the Northeast region–74.30/100,000 in the Southeast region of the country (Brazil, 2016). Moreover, breast cancer mortality is much higher in Brazil than in most high‐income countries, with mortality rising from 10.83/100,000 in 2002 (Carioli et al., 2018)–14.5/100,000 in 2018 (International Agency for Research on Cancer, 2020). This contrasts with most European and North American countries where mortality has declined, largely attributed to treatment advances as well as early cancer detection via mammography screening (Wild et al., 2020).

Mammography screening is considered the gold standard for the early detection of breast cancer because smaller lesions can be identified and treatment initiated earlier in the disease trajectory, thereby improving treatment effectiveness (Silva & Hortale, 2012). The Brazilian Ministry of Health established guidelines in 2004, which were updated in 2015 by the Brazilian National Institute of Cancer (Brazil, 2015), to now recommend that all women aged 50–69 years undergo mammography screening every 2 years. Women aged 40–49 years are advised to undergo mammography screening only if they are deemed to be at high risk for breast cancer or if their annual clinical breast examination is abnormal. Specific legislation to ensure access to mammography was enacted in 2008. Despite the recommendation and legislation, Brazilian data indicate that overall, many women are not undergoing mammography screening, particularly those aged 50–60 years. Furthermore, Brazilian research suggests that many women are diagnosed at an advanced stage, resulting in reduced likelihood of cure as well as more costly treatments (Lee et al., 2012).

Women's non‐adherence to mammography screening has been the focus of research worldwide, particularly in western countries. Factors found to be associated with mammography non‐adherence include lower educational attainment, lower individual and community socio‐economic status, non‐White ethnicity and increased presence of co‐morbid disease (Hubbard et al., 2016). Recent reviews also suggest that prior breast and cervical cancer screening behaviour predicts mammography use, as does access to a physician, a physician recommendation, care by an obstetrician/gynaecologist and having health insurance and a regular source of health care (Madadi, 2014; Sarma, 2015). Social factors, such as a lack of social support and cultural norms of privacy and modesty, may also influence women's screening behaviour (Sarma, 2015). Though this research provides insight, findings might have limited applicability to the unique Brazilian context.

Various Brazilian studies have investigated mammography adherence (Moreira et al., 2018); however, there has been no knowledge synthesis that identifies the factors related to adherence across studies. An overall understanding of the factors that influence Brazilian women's use of mammography is foundational to identifying gaps in the literature, so as to inform future research endeavours as well as the development of effective health services that can create the conditions that promote adherence. Accordingly, the purpose of this scoping review was to identify the factors related to mammography screening adherence among women in Brazil.

## 2 METHODS

### 2.1 Design

A scoping review was deemed appropriate because this type of review is used to address an exploratory question with the aim of mapping the key concepts, types of evidence and gaps in research related to a defined area (Colquhoun et al., 2014). We employed the methodological framework outlined by Arksey and O’Malley (2005) and enhanced by Levac and colleagues (2010), which included the stages of: (a) identifying the research question; (b) identifying relevant studies; (c) study selection; (d) charting the data; and (e) collating, summarizing and reporting the results. We did not conduct the optional stage of consultation with stakeholders (Arksey & O’Malley, 2005; Colquhoun et al., 2014; Levac et al., 2010). The research question for this scoping review was: What factors have been investigated and found to be related to mammography screening adherence among women in Brazil?

### 2.2 Identifying relevant studies: search strategy

We built the literature search strategy in consultation with a medical librarian and searched 11 databases: MEDLINE (through Ovid), PubMed, Web of Science, CINAHL (through EBSCOhost), Elsevier ScienceDirect, LILACS (through BVS), SciELO, Cancerlit, BDEnf (through BVS), MedCarib (through BVS) and PAHO (through BVS). The general search terms included mammography and Brazil (see the File S1 for specific search terms used), and we limited our searches to studies published between 2006–January 2020 (the date of our final search). The year 2006 was chosen as a starting point because of the country‐wide institutional reforms focussed on women's health that were established that year (Brazil, 2015). All searches were run consecutively on the same day.

### 2.3 Study selection

Studies included in this review (a) were published in English, Spanish or Portuguese; (b) were published in a peer review journal; (c) had a study sample that included women in Brazil; (d) investigated factors related to mammography screening; (e) measured mammography screening adherence among individuals or groups; (f) included the outcome of mammography screening as self‐reported or collected via a health service database; and (g) used a comparative research design. We excluded studies that were published in the grey literature in the form of reports, book chapters, conference papers or theses.

Two independent reviewers (CM and AM) performed the initial title and abstract screening of the articles and the articles that did not meet the inclusion criteria were excluded. The full text of the remaining articles were retrieved and screened according to the inclusion criteria. Where there was ambiguity, FH and *SD* assessed the article to determine the final set of studies to be included in this review. We also reviewed the reference lists of relevant manuscripts, but no additional publications were included.

### 2.4 Charting the data: data extraction

We adapted the EPPI‐Centre systematic reviews instrument (Newman & Elbourne, 2004) to extract data from the included studies based on the purpose of our review. Using our data extraction template, we retrieved the following information from each study: author, publication year, language, study design, setting and sample, mammography adherence (%), factors related to mammography adherence and non‐significant factors examined.

### 2.5 Collating and summarizing: data analysis and quality assessment

We divided the selected studies into three groups according to the outcome used in the included studies: (a) adherence to mammography within 2 years (as per national recommendations); (b) never versus ever had mammography; or (c) adherence to mammography at other time points. We then identified all the factors evaluated in the studies and grouped these factors into the following: demographic, socio‐economic, health service use, medical and health history and previous cancer screening. For each study, we identified which factors were found to be significantly related to mammography adherence and whether these findings were obtained through bivariate or multivariate analysis.

Although study quality was not a criterion for inclusion in our review, we used the UK Government Social Research Service (GSRS) weight of evidence appraisal tool (Gough, 2007) to appraise the quality of the included studies. The GSRS appraisal tool assesses the trustworthiness of the findings, the appropriateness of the design and analysis and the relevance of the focus of the study for addressing the questions of the review. Each of the three sections was scored separately and then summed to yield assessments of low‐, medium‐ or high‐quality evidence. Two reviewers (CM and AM) independently assessed each article. English‐language articles were also assessed by FH and VSD. When there were differences in scores, the reviewers discussed the rationale for their scores and came to agreement.

### 2.6 Ethics

Ethical approval was not required for this study.

## 3 RESULTS

### 3.1 Identification and selection of studies

**FIGURE 1:**
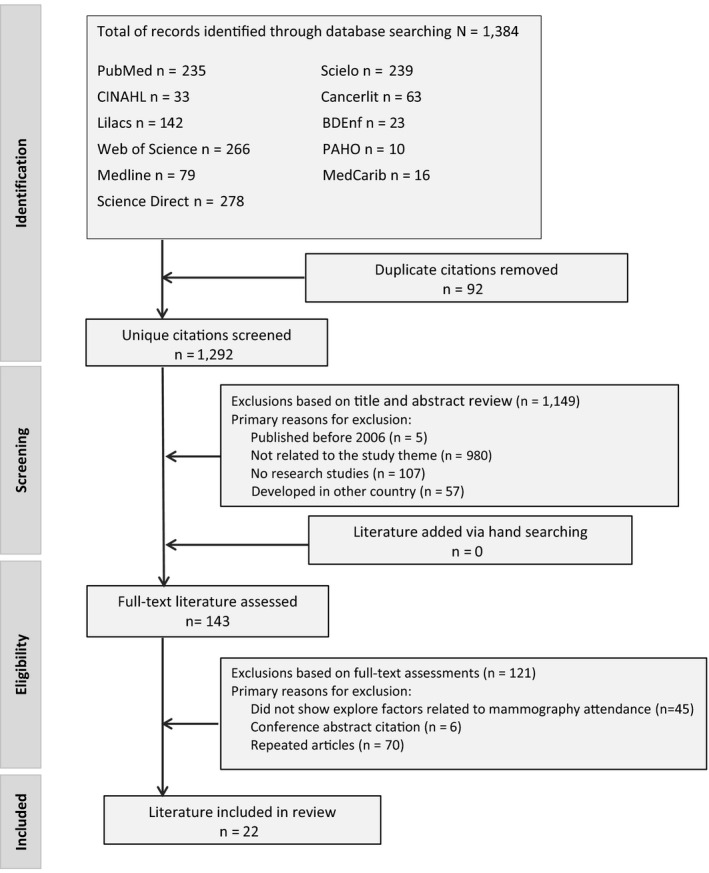
PRISMA flow diagram showing article selection

We identified a total of 1,384 articles from our initial search of the 11 databases. We then excluded 92 duplicates and 1,288 articles that did not meet the inclusion criteria during the title and abstract screening. We reviewed the full text of 143 articles to determine whether they met the inclusion criteria. At the end of the identification and selection process, a final sample of 22 studies met all inclusion criteria and were retained for data extraction (Figure 1).

### 3.2 Characteristics of studies

The publication dates of the 22 studies included in this scoping review ranged from 2006–2019, with 50% of the studies published after 2014. All 22 studies used quantitative, non‐experimental methods, wherein 2 were longitudinal (Caleffi et al., 2010; Marchi & Gurgel, 2010) and the remainder were cross‐sectional. All studies assessed the relationship of various factors to mammography adherence. Data were collected from pre‐existing Brazilian National Health surveys (9 studies), women in a health unit/centre (7 studies), women via a home interview (5 studies) and women via telephone (1 study). Eleven studies assessed adherence to mammography within 2 years (as per national recommendations), two of which divided their sample into 2 age groups and conducted separate analyses and one of which divided their sample into two different years. Six studies assessed never versus ever had mammography, one of which divided their sample into two different years, while another into 2 different regions of Brazil and conducted separate analyses. The five studies that assessed other frequencies of mammography included one study that analysed the data from two different age groups separately. Thus, among the 22 studies, there were 27 separate investigations.

Among the 22 studies, data from each of the five official regions of Brazil were included and nine of the studies collected data from two or more regions (Borges et al., 2016; Lima‐Costa & Matos, 2007; Malta & Bernal, 2014; Melo et al., 2016; Novaes et al., 2006; Oliveira et al., 2011; Rodrigues et al., 2015; Theme Filha et al., 2016; Viacava et al., 2019). Nine of the studies had sample sizes greater than 10,000 (Borges et al., 2016; Lima‐Costa & Matos, 2007; Malta & Bernal, 2014; Novaes et al., 2006; Oliveira et al., 2011; Rodrigues et al., 2015; Theme Filha et al., 2016; Viacava et al., 2019; Vieira et al., 2015), with the smallest sample being 40 women (Moreira et al., 2018). Five studies included women less than 40 years of age (Bim, 2010; Marchi & Gurgel, 2010; Marchi et al., 2006; Novaes et al., 2006; Oliveira et al., 2011). Across studies, mammography adherence ranged from 15.6% (Rodrigues et al., 2015)–97.1% (Brum et al., 2018); however, it should be noted that the sample for the Brum et al., (2018) study consisted of women at high risk for breast cancer who were attending a university hospital. There was no chronological pattern to the rates and most studies showed mammography adherence rates of <50%. Study characteristics, including the weight of evidence scores, are summarized in Table 1. Of the 27 investigations, their weight of evidence scores was distributed as follows: 4 high; 17 medium; and 6 low.

**TABLE 1:** Characteristics of studies measuring mammography adherence, by time frame of mammography uptake

Study code	Author (publication year/language)	Setting and sample			Mammography adherence	Factors related to mammography adherence	Non‐significant factors examined	GSRS scores
a) Had mammography during the last 2 years (national guidelines)								
1	Novaes et al., (2006)/Portuguese			National *N* = 107,094 Ages: 25–80 years	63.9%	Multivariate analysis results: *Demographic:* Age [50–59 years old], Urban/rural household location [urban] *Socio‐economic:* Education [high], Income [high], Health insurance [private] *Health service use:* Medical consult in last 15 days [yes], Type of health service used in last 15 days [Private]. *Medical and health history:* Self‐reported heath [good/very good].	Marital status Employment Number of children	Medium
2A	Lima‐Costa and Matos (2007)/Portuguese^a^			National *N* = 16,570 Ages: 50–59 years	46.3%	Multivariate analysis results: *Demographic:* Urban/rural household location [urban], Region of Brazil [Southeast] *Socio‐economic:* Education [≥8 years], Income [high], Health insurance [private] *Health service use:* Number of medical consults in prior 12 months [≥1] *Previous cancer screening:* Previous Pap smear [≥3 years ago]	Cohabiting with family members Self‐reported heath Difficulty performing activities of daily living Chronic disease	Medium
2B	Lima‐Costa and Matos (2007)/Portuguese^a^			National *N* = 10,722 Ages: 60–69 years	36.9%	Multivariate analysis results: *Demographic:* Urban/rural household location [urban], Region of Brazil [Southeast] *Socio‐economic:* Education [≥8 years], Income [high], Health insurance [private] *Health service use:* Number of medical consults in prior 12 months [≥1] *Medical and health history:* Self‐reported heath [good/very good] *Previous cancer screening:* Previous Pap Smear [≥3 years ago]	Cohabiting with family members Difficulty performing activities of daily living Chronic disease	Medium
3	Marinho et al., (2008)/Portuguese and English			Southeast Region *N* = 663 Ages: ≥40 years	35.7%	Bivariate analysis results: *Socio‐economic:* Income [high], Employment [paid job]	Age Education Marital status Waiting time at health centre	Medium
4	Marchi and Gurgel (2010)/Portuguese	Southeast Region *N* = 460 Ages: ≥40 years			29.8%	Bivariate analysis results: *Demographic:* Age [50–59 years old] *Socio‐economic:* Health insurance [private] *Health service use: M*edical consult in last 12 months [yes] *Medical and health history:* Menopause [yes] *Previous cancer screening:* Previous mammography [24 months ago], Follow up with specialist [yes]	Education Marital status Employment Income Knowledge about mammogram Presence of breast disease Family history of breast cancer	Medium
5	Malta and Bernal (2014)/Portuguese	National *N* = 54,099 Ages: 50–69 years			N/A	Multivariate analysis results: *Socio‐economic:* Education [≥12 years], Health insurance [private]	–	Medium
6	Theme Filha et al., (2016)/English	National *N* = 11,212 Ages: 40–69 years			41.5%	Multivariate analysis results: *Demographic:* Marital status [living with a partner], Urban/rural household location [urban], Region of Brazil [Midwest] *Socio‐economic:* Education [ ≥11 years], Health insurance [private] *Medical and health history:* Tobacco use [no], Physical activity [yes], Recommended fruit and vegetable consumption [yes]	Race Self‐rated heath	High
7	Souza et al., (2017)/Portuguese and English	North Region *N* = 241 Ages: 40–69 years			44.4%	Multivariate analysis results: *Socio‐economic:* Education [high] *Health service use:* Medical consult in past 12 months [yes], Health agent home visit [yes]	Age Health insurance Income Government financial aid	High
8	Moreira et al., (2018)/Portuguese and English	Northeast Region *N* = 40 Ages: 50–69 years			N/A	Bivariate analysis results: *Demographic:* Age [65–69 years old], Marital status [married/stable union], Number of children [1–2 children] *Socio‐economic:* Education [high], Income [high] *Medical and health history:* Menopause [no], Early Menarche [no], Personal history of cancer [no], Family history of cancer [yes]	Race Urban/rural household location Employment History of hormone replacement therapy	Medium
9	Brum et al., (2018)/Portuguese and English	Southeast Region *N* = 820 Ages 20–69 *N* = 27 Ages: 50–69 years			97.1%	Bivariate analysis results: –	Family history of cancer Knows someone with a history of cancer	Low
10	Buranello et al., (2018)/Portuguese and English	Southeast Region *N* = 1,512 Ages: ≥20 years *N* = 511 ≥50 years			68.6% 50–69 years 46.2% ≥70 years	Multivariate analysis results: *Demographic:* Age [younger]. *Socio‐economic:* Income [high], Health insurance [public].	Race Marital status Matriarch head of family Education Benign breast lumps Personal history of breast cancer Personal history of cancer Tobacco use Body mass index Physical activity	Medium
11A	Viacava et al., (2019)/Portuguese and English^b^	National *N* = 16,360 Age: 50–69 years			54.2%	Multivariate analysis results: *Socio‐economic:* Education [≤3 years].	–	Low
11B	Viacava et al., (2019)/Portuguese and English^b^	National *N* = 52,882 Age: 50–69 years			60.0%	Multivariate analysis results: *Socio‐economic:* Education [≤3 years].	–	Low
b) Had mammography: Ever versus never								
12	Marchi et al., (2006)/Portuguese)		Southeast Region *N* = 643 Ages: ≥30 years		60.9%	Bivariate analysis results: *Demographic:* Age [≥50 years old] *Socio‐economic:* Health insurance [public]	Age at first mammogram	Low
13	Bim et al., (2010)/Portuguese		South Region *N* = 885 Ages: 18–86 years		24.2%	Bivariate analysis results: *Demographic:* Age [39–48 years old] *Socio‐economic:* Income [high]	–	Low
14A	Oliveira et al., (2011)/Portuguese^b^	National (2003) *N* = 49,619,835 Ages: ≥25 years			42.5%	Multivariate analysis results: *Demographic:* Age [50–69 years old], Marital status [lives with partner], Urban/rural household location [urban], Region of Brazil [Southeast] *Socio‐economic:* Education [high level], Income [high], Health insurance [private] *Health Service Use:* Previous medical appointment [in past 12 months]	Race Self‐rated health Location of mammogram clinic	Medium
14B	Oliveira et al., (2011)/Portuguese^b^	National (2008) *N* = 57,357,243 Ages: ≥25 years			54.8%	Multivariate analysis results: *Demographic:* Age [50–69 years old], Marital status [lives with partner], Urban/rural household location [urban], Region of Brazil [Southeast] *Socio‐economic:* Education [high level], Income [high], Health insurance [private] *Health service use:* Previous medical appointment [past 12 months]	Race Self‐rated health Location of mammogram clinic	Medium
15	Vieira et al., (2015)/English	Southeast Region *N* = 54,238 Ages: 40–69 years			19.2%	Multivariate analysis results: *Demographic:* Age [50–59 years old] *Socio‐economic:* Education [high level], Income [high] *Previous cancer screening:* Location of mammogram [Hospital], Influenced by people/programme [Doctor]	–	High
16	Melo et al., (2016)/English	National *N* = 11,607,000 Ages: ≥40 years			N/A	Multivariate analysis results: *Demographic:* Age [50–69 years old], Race [yellow] *Socio‐economic:* Education [≥15 years], Income [high], Health insurance [private]	–	Medium
c) Had mammography: Other time frames								
17A	Borges et al., (2016)/English^c^	National/Northeast *N* = 17,681 Ages: 40–69 years			68.0%	Multivariate analysis results: *Demographic:* Age [50–59 years old] *Socio‐economic:* Education [9–11 years], Income [high]	Race Marital status	Medium
17B	Borges et al., (2016)/English)^c^	National/South *N* = 10,037 Ages: 40–69 years			82.5%	Multivariate analysis results: *Demographic:* Age [50–59 years old] *Socio‐economic:* Education [9–11 years], Income [high]	Race Marital status	Medium
18	Caleffi et al., (2010)/English	South *N* = 3,749 Ages: 40–69 years			57.6% (≤18 months)	Multivariate analysis results: *Demographic:* Number of children [few] *Socio‐economic:* Education [>8 years] *Medical and health history:* High genetic risk [yes], History of hormone replacement therapy [yes], History of oral contraceptive use [yes], Previous tobacco use [yes]. Current tobacco use [no].	Age Body mass index Regular breast self‐examination Previous breast biopsy Recruitment by research team or clinic health providers	High
19A	Schneider et al., (2014)/Portuguese^a^			South *N* = 447 Ages: 40–59 years	42.9% (annually)	Multivariate analysis results: *Socio‐economic:* Health insurance [private]	Age Race Marital status Education Income Employment	Medium
19B	Schneider et al., (2014)/Portuguese^a^			South *N* = 510 Ages: 60–69 years	37.3% (annually)	Multivariate analysis results: *Demographic:* Marital status [lives with partner] *Socio‐economic:* Education [9–11 years], Income [high]	Race Employment Health insurance	Medium
20	Oliveira et al., (2014)/Portuguese and English	Southeast *N* = 255 Ages: ≥60 years			24.3% (<1 year) 28.6% (1–3 years) 22.3% (>3 year)	Bivariate analysis results: *Demographic:* Race [White]	–	Medium
21	Rodrigues, Cruz, and Paixão (2015)/Portuguese	National *N* = 67.511 Ages: 40–108 years			35.0% (<1 year) 16.5% (1–2 years) 15.6% (>2 years)	Multivariate analysis results: *Demographic:* Age [older], Race [White], Marital status [lives with spouse], Cohabiting with family members [no child], Urban/rural household location [urban], Region of Brazil [South/Southeast] *Socio‐economic:* Education [high], Income [high], Health insurance [private] *Medical and health history:* Self‐rated health [positive], Personal history of cancer [yes], Tobacco use [no].	Has child <14 years	Low
22	Lopes et al., (2016)/English	South *N* = 525 Ages: ≥40 years			54.1% (annually)	Bivariate analysis results: *Demographic:* Age [60–69 years old] *Medical and health history:* Past hormone replacement therapy [yes], Past use of oral contraceptive [yes] *Previous cancer screening:* Previous clinical breast examination [no], Performs breast self‐examination [no].	Education Marital status Race Employment Self‐rated health Personal history of cancer Menopause Current hormone replacement therapy Age at menarche Parity Breastfeeding Family history of breast cancer Tumour characteristics	Medium

Abbreviations: GSRS scores, UK Government Social Research Service weight of evidence appraisal tool; N/A, not available.

^a^ This study divided participants into two groups of different age ranges for analysis.

^b^ This study collected data in 2003 and 2008, and reported findings by year of data collection.

^c^ This study grouped participants into two subsamples by region of Brazil (South and Northeast) for analysis.

### 3.3 Demographic and socio‐economic factors

Table 2 summarizes the results of the 27 investigations of factors related to mammography adherence. The results are grouped by category of factors. Wherever possible, multivariate results are reported, as indicated on the table.

**TABLE 2:** Summary of factors associated with mammography adherence

Timing of outcomes		National recommendation (within the last 2 years)													Never versus ever								Other time frames					
Factors	Study code and type of analysis	1 M	2A M	2B M	3 B	4 B	5 M	6 M	7 M	8 B	9 B	10 M	11A M	11B M	12 B	13 B	14A M	14B M	15 M	16 M	17A M	17B M	18 M	19A M	19B M	20 B	21 M	22 B
Demographic factors	1. Age	S	‐	‐	ns	S	‐	‐	ns	S	‐	S	‐	‐	S	S	S	S	S	S	S	S	ns	ns	‐	‐	S	S
	2. Race	‐	‐	‐	‐	‐	‐	ns	‐	ns	‐	ns	‐	‐	‐	‐	ns	ns	‐	S	ns	ns	‐	ns	ns	S	S	ns
	3. Marital status	ns	‐	‐	ns	ns	‐	S	‐	S	‐	ns	‐	‐	‐	‐	S	S	‐	‐	ns	ns	‐	ns	S	‐	S	ns
	4. Matriarch head of family	‐	‐	‐	‐	‐	‐	‐	‐	‐	‐	ns	‐	‐	‐	‐	‐	‐	‐	‐	‐	‐	‐	‐	‐	‐	‐	‐
	5. Cohabiting with family	‐	ns	ns	‐	‐	‐	‐	‐	‐	‐	‐	‐	‐	‐	‐	‐	‐	‐	‐	‐	‐	‐	‐	‐	‐	S	‐
	6. Number of children	ns	‐	‐	‐	‐	‐	‐	‐	S	‐	‐	‐	‐	‐	‐	‐	‐	‐	‐	‐	‐	S	‐	‐	‐	‐	‐
	7. Urban/rural household location	S	S	S	‐	‐	‐	S	‐	ns	‐	‐	‐	‐	‐	‐	S	S	‐	‐	‐	‐	‐	‐	‐	‐	S	‐
	8. Region of Brazil	‐	S	S	‐	‐	‐	S	‐	‐	‐	‐	‐	‐	‐	‐	S	S	‐	‐	‐	‐	‐	‐	‐	‐	S	‐
Socio‐economic factors	9. Education	S	S	S	ns	ns	S	S	S	S	‐	ns	S	S	‐	‐	S	S	S	S	S	S	S	ns	S	‐	S	ns
	10. Income	S	S	S	S	ns	‐	‐	ns	S	‐	S	‐	‐	‐	S	S	S	S	S	S	S	‐	ns	S	‐	S	‐
	11. Employment	ns	‐	‐	S	ns	‐	‐	‐	ns	‐		‐	‐	‐	‐	‐	‐	‐	‐	‐	‐	‐	ns	ns	‐	‐	ns
	12. Private health insurance	S	S	S	‐	S	S	S	ns	‐	‐	S	‐	‐	S	‐	S	S	‐	S	‐	‐	‐	S	ns	‐	S	S
	13. Government financial aid	‐	‐	‐	‐	‐	‐	‐	ns	‐	‐	‐	‐	‐	‐	‐	‐	‐	‐	‐	‐	‐	‐	‐	‐	‐	‐	‐
Health service use	14. Previous medical consult	S	S	S	‐	S	‐	‐	S	‐	‐	‐	‐	‐	‐	‐	S	S	‐	‐	‐	‐	‐	‐	‐	‐	‐	‐
	15. Health agent home visit	‐	‐	‐	‐	‐	‐	‐	S	‐	‐	‐	‐	‐	‐	‐	‐	‐	‐	‐	‐	‐	‐	‐	‐	‐	‐	‐
	16. Type of health service	S	‐	‐	‐	‐	‐	‐	‐	‐	‐	‐	‐	‐	‐	‐	‐	‐	‐	‐	‐	‐	‐	‐	‐	‐	‐	‐
Medical and health history	17. Menopause	‐	‐	‐	‐	S	‐	‐	‐	S	‐	‐	‐	‐	‐	‐	‐	‐	‐	‐	‐	‐	‐	‐	‐	‐	‐	ns
	18. Early Menarche	‐	‐	‐	‐	‐	‐	‐	‐	S	‐	‐	‐	‐	‐	‐	‐	‐	‐	‐	‐	‐	‐	‐	‐	‐	‐	‐
	19. Self‐reported heath	S	ns	S	‐	‐	‐	ns	‐	‐	‐	‐	‐	‐	‐	‐	‐	‐	‐	‐	‐	‐	‐	‐	‐	‐	S	ns
	20. Difficulty performing ADL	‐	ns	ns	‐	‐	‐	‐	‐	‐	‐	‐	‐	‐	‐	‐	‐	‐	‐	‐	‐	‐	‐	‐	‐	‐	‐	‐
	21. Chronic disease	‐	ns	ns	‐	‐	‐	‐	‐	‐	‐	‐	‐	‐	‐	‐	‐	‐	‐	‐	‐	‐	‐	‐	‐	‐	‐	‐
	22. Benign breast lumps	‐	‐	‐	‐	‐	‐	‐	‐	‐	‐	ns	‐	‐	‐	‐	‐	‐	‐	‐	‐	‐	‐	‐	‐	‐	‐	‐
	23. High genetic risk	‐	‐	‐	‐	‐	‐	‐	‐	‐	‐	‐	‐	‐	‐	‐	‐	‐	‐	‐	‐	‐	S	‐	‐	‐	‐	‐
	24. Personal history of breast cancer	‐	‐	‐	‐	‐	‐	‐	‐	‐	‐	ns	‐	‐	‐	‐	‐	‐	‐	‐	‐	‐	‐	‐	‐	‐	‐	‐
	25. Personal history of cancer	‐	‐	‐	‐	‐	‐	‐	‐	S	‐	ns	‐	‐	‐	‐	‐	‐	‐	‐	‐	‐	‐	‐	‐	‐	S	ns
	26. Family history of cancer	‐	‐	‐	‐	‐	‐	‐	‐	S	ns	‐	‐	‐	‐	‐	‐	‐	‐	‐	‐	‐	‐	‐	‐	‐	‐	ns
	27. Knows someone with a history of cancer	‐	‐	‐	‐	‐	‐	‐	‐	‐	ns	‐	‐	‐	‐	‐	‐	‐	‐	‐	‐	‐	‐	‐	‐	‐	‐	‐
	28. Past use of HRT	‐	‐	‐	‐	‐	‐	‐	‐	‐	‐	‐	‐	‐	‐	‐	‐	‐	‐	‐	‐	‐	S	‐	‐	‐	‐	S
	29. Current use of HRT	‐	‐	‐	‐	‐	‐	‐	‐	ns	‐	‐	‐	‐	‐	‐	‐	‐	‐	‐	‐	‐	‐	‐	‐	‐	‐	ns
	30. Past use of oral contraceptives	‐	‐	‐	‐	‐	‐	‐	‐	‐	‐	‐	‐	‐	‐	‐	‐	‐	‐	‐	‐	‐	S	‐	‐	‐	‐	S
	31. Tobacco use	‐	‐	‐	‐	‐	‐	S	‐	‐	‐	ns	‐	‐	‐	‐	‐	‐	‐	‐	‐	‐	S	‐	‐	‐		‐
	32. Body mass index	‐	‐	‐	‐	‐	‐	‐	‐	‐	‐	ns	‐	‐	‐	‐	‐	‐	‐	‐	‐	‐	‐	‐	‐	‐	‐	‐
	33. Physical activity level	‐	‐	‐	‐	‐	‐	S	‐	‐	‐	ns	‐	‐	‐	‐	‐	‐	‐	‐	‐	‐	‐	‐	‐	‐	‐	‐
	34. Fruit & vegetable consumption	‐	‐	‐	‐	‐	‐	S	‐	‐	‐	‐	‐	‐	‐	‐	‐	‐	‐	‐	‐	‐	‐	‐	‐	‐	‐	‐
Previous cancer screening	35. Previous mammogram	‐	‐	‐	‐	S	‐	‐	‐	‐	‐	‐	‐	‐	‐	‐	‐	‐	‐	‐	‐	‐	‐	‐	‐	‐	‐	‐
	36. Location of mammogram clinic	‐	‐	‐	‐	‐	‐	‐	‐	‐	‐	‐	‐	‐	‐	‐	‐	‐	S	‐	‐	‐	‐	‐	‐	‐	‐	‐
	37. Previous clinical breast examination	‐	‐	‐	‐	‐	‐	‐	‐	‐	‐	‐	‐	‐	‐	‐	‐	‐	‐	‐	‐	‐	‐	‐	‐	‐	‐	S
	38. Performs breast self‐examination	‐	‐	‐	‐	‐	‐	‐	‐	‐	‐	‐	‐	‐	‐	‐	‐	‐	‐	‐	‐	‐	ns	‐	‐	‐	‐	S
	39. Follow up with specialist	‐	‐	‐	‐	S	‐	‐	‐	‐	‐	‐	‐	‐	‐	‐	‐	‐	‐	‐	‐	‐	‐	‐	‐	‐	‐	‐
	40. Influenced by people/programme	‐	‐	‐	‐	‐	‐	‐	‐	‐	‐	‐	‐	‐	‐	‐	‐	‐	S	‐	‐	‐	‐	‐	‐	‐	‐	‐
	41. Previous Pap smear	‐	S	S	‐		‐	‐	‐	‐	‐	‐	‐	‐	‐	‐	‐	‐	‐	‐	‐	‐	‐	‐	‐	‐	‐	‐

Note

‐ not assessed.

Abbreviations: ADL, activities of daily living; B, bivariate; HRT, hormone replacement therapy; M, multivariate; ns, non‐significant; S, significant.

Demographic and socio‐economic factors were the most commonly investigated and within these categories, age, race, marital status, education, income and health insurance were the most frequently assessed. Older age was related to mammography adherence in all but five of the 18 investigations that included age (6 bivariate, 12 multivariate analyses). The study by Buranello et al., (2018) was the only study to find declining rates of adherence among older participants after controlling for other factors. Race was not found to be related to mammography adherence, except in three of the 13 investigations (3 bivariate, 10 multivariate). Only six of the 14 investigations that examined the association between marital status and mammography adherence found significant results, with higher rates of adherence among women living with partners (4 bivariate, 10 multivariate). Higher education was found to be associated with adherence in 13 of the 18 investigations that included education (4 bivariate, 14 multivariate); the other five investigations yielded non‐significant results. Similarly, higher income (18 investigations) and health insurance (16 investigations) were consistently associated with adherence. The exceptions were the three and two studies, for income and health insurance, respectively, that found non‐significant results. Though less commonly assessed, when urban/rural household location (8 investigations) and region (6 investigations) were included in multivariate analysis, urban residence and living in the southeast of Brazil were significantly related to higher levels of mammography adherence.

### 3.4 Health service use factors

A previous medical appointment was significantly related to mammography adherence in all seven investigations where this factor was included, despite variation in time frame ranging from 15 days (Novaes et al., 2006)–12 months (Lima‐Costa & Matos, 2007; Marchi & Gurgel, 2010; Oliveira et al., 2011; Souza et al., 2017). These previous medical appointments included consultations with a nurse or primary care provider (Lima‐Costa & Matos, 2007; Novaes et al., 2006; Oliveira et al., 2011; Souza et al., 2017), or specialists such as a gynaecologist or oncologist (Marchi & Gurgel, 2010).

### 3.5 Medical and health history

Eighteen medical and health history factors were examined. Self‐reported health was the most commonly investigated, with a positive perception of health found to be associated with mammography adherence in three of the six investigations (Lima‐Costa & Matos, 2007; Novaes et al., 2006; Rodrigues et al., 2015). Thirteen of the other medical and health history factors were assessed in only one or two investigations. Of those that were investigated more than twice, mixed results were found for menopause (3 investigations), personal history of cancer (4 investigations), family history of cancer (3 investigations) and tobacco use (3 investigations). No association was found for difficulties in performing daily activities, chronic disease, benign breast lumps, personal history of breast cancer, knowing someone with a history of breast cancer, current use of HRT or body mass index, although each of these was assessed in only 1 or 2 investigations.

### 3.6 Previous cancer screening

Seven factors related to cancer screening were investigated. Although each factor was only included in one or two investigations, all were significant when included, with the exception of breast self‐examination in one investigation (Caleffi et al., 2010).

## 4 DISCUSSION

To our knowledge, this is the first knowledge synthesis of the published literature to identify factors related to mammography adherence among women in Brazil. We located 22 studies, representing 27 separate investigations, wherein adherence was measured by: (a) whether women followed national recommendations (11 studies); (b) ever had a mammogram (6 studies); or (c) had a mammogram within another time frames (5 studies). Only two studies were longitudinal, with the remainder using a cross‐sectional design with its risk of recall bias.

Demographic and socio‐economic factors were the most commonly investigated, with older age, urban residence, living in the southeast of Brazil, higher level of education, higher income and private health insurance most consistently associated with mammography adherence. The association with previous health service use, medical and health history and previous cancer screening practices was investigated less often and with mixed results. One exception was the consistently positive relationship found between a recent previous medical appointment and mammography adherence in seven investigations.

Mammography adherence ranged widely across investigations, but the study samples varied from high risk samples (Buranello et al., 2018) to nationally representative samples (e.g. Viacava et al., 2019). However, the wide range in mammography adherence rates across studies also raises questions about differential access to mammography screening. There are large regional variations in health and health services in Brazil, including access to primary and speciality care (Albuquerque et al., 2017). Brazil comprises 26 states and the Federal District, grouped into five macro regions: north, northeast, centre west, southeast and south. The south and southeast are the richest and most developed; these are the two regions that have long shown the longest life expectancy for both males and females (Borges, 2017). A recent analysis by Albuquerque and colleagues (2017) showed marked differences in the number of doctors and hospitals per 1,000 inhabitants by level of socio‐economic development in the area. In 2016, the least developed areas of Brazil had 0.63 doctors and 1.7 hospital beds per 1,000 inhabitants, compared with 2.61 doctors and 2.5 hospital beds in the most urbanized and industrialized areas. A further analysis by Andrade and colleagues (2018) showed a positive relationship between the supply of doctors in a region and uptake of the Family Health Strategy, a primary healthcare programme. Thus, it is not unreasonable to assume that access to mammography screening also varies by region—consistent with the findings of this scoping review that showed that living in the southeast was associated with greater uptake of mammography screening.

Findings from the reviewed studies that investigated the influence of socio‐economic factors at the individual level suggest that those who are more highly educated and have higher incomes and private health insurance are more likely to have a mammogram. The association of higher socio‐economic status with mammography adherence, as well as other types of health screening, has been well documented in other developing as well as developed countries. For example, data from the Korean National Health and Nutrition Examination Survey showed that individuals with a lower socio‐economic status were less likely to have had a comprehensive health check‐up within the prior 2 years (Shin et al., 2018). The relationships between socio‐economic status and mammography update may be due to differences in access to information or perceived need, as well as economic barriers. For example, a study by Donnelly and colleagues (2015) of breast cancer screening in Qatar found that higher education and higher income were not only the strongest predictors of mammogram screening, but were also strongly associated with greater awareness of the national screening guidelines. Similarly, a recent study by de Oliveira et al., (2018) of women living in a rural area of Brazil found that both income and education levels were associated with knowledge and attitudes to breast cancer screening. Thus, even though women in Brazil have access to publicly funded healthcare services including mammography screening, there may still be barriers related to socio‐economic status. Other barriers related to socio‐economic status may include access to transportation or the opportunity to leave work for a medical appointment (Shin et al., 2018). For example, a study of barriers to the use of breast cancer screening services in Nigeria found that 66.5% of the women reported transportation difficulties (Okoronkwo et al., 2015). Brazilian researchers have also commented on the relationship between income and women's ability to manage their own time (Melo et al., 2016). Finally, it should be noted that although several studies showed significant results for race in bivariate analyses, the relationships generally became non‐significant in multivariate analyses (e.g. Buranello et al., 2018; Oliveira et al., 2011; Theme Filha et al., 2016), indicating that the socio‐economic conditions associated with race are the primary contributor to non‐adherence. This reinforces the importance of multivariate analysis, controlling for other important factors.

Another reason for the importance of socio‐economic status may be that there is competition for limited screening resources when most women are dependent on publicly provided health services (Vieira, 2015). This may help explain the importance of private insurance as a predictor of mammogram uptake in Brazil. Also, as discussed above, health services do vary by region of Brazil and several studies using multivariate analyses have shown that region is predictive of mammography uptake, even after controlling for the individual's socio‐economic status. Until 2000, the Standardized World Income Inequality Database showed that Brazil ranked as one of the most unequal countries in the world (Solt, 2016). Although there have been improvements, Brazil still shows marked regional differences and inequalities in income and other social conditions (Melo et al., 2016).

The findings of our scoping review suggest that further research is required to tease apart the ways socio‐economic factors influence adherence to mammography screening guidelines, including studies that move beyond investigations at the individual level to investigate the mechanisms by which structural barriers influence mammography uptake. For example, in addition to assessing the overall availability of health services, it is also important to account for the perceived quality of services. Studies have shown that there is a need to strengthen the primary healthcare centres in Brazil, not just in terms of the physical condition of the facilities, but also with respect to the quality of care. For example, Fausto and colleagues (2017) identified challenges related to the continuity of care between the primary healthcare centres and other health services, evidenced by variations in referral patterns to specialists or for examinations, as well as variations in recommendations for follow up appointments. Studies in other developing countries have also found that poor service, limited time with clinicians, shortages of clinic supplies, the distance and time required to travel to the clinics and waiting times after reaching the clinic were the main barriers to accessing health services (Legido‐Quigley et al., 2019).

Future research should also consider factors that were not investigated in the studies in this review but may be influential. For example, the success of cancer screening programmes is at least partially dependent on individual and public health education to raise awareness about cancer and the benefits of early detection (Sivaram et al., 2018). Therefore, the receipt or recall of patient education or public health messaging about breast cancer screening should be assessed at the individual level. However, it should also be assessed at the community and policy levels, as various regions may have different policies and practices regarding public health messaging about mammography screening. Other factors that have been found to be associated with mammogram adherence but were not investigated in the studies included in this review include the influence of religion, discomfort/pain experienced during a previous mammogram, fear of a cancer diagnosis and embarrassment (Padela et al., 2015; Sousa, 2014).

### 4.1 Quality of evidence

The quality of evidence varied across studies. Most of the 27 investigations were rated as medium quality, with only six being rated as low quality. Most studies drew on data from national or regional health surveys that were designed for a broader purpose. All but two of the investigations used cross‐sectional designs with the potential for recall bias. Eight of the investigations were also weakened by the sole use of bivariate analysis and none reported effect sizes. Although a lack of detail in many studies created challenges for assessing the quality of the evidence, our assessment suggests that the set of studies included in this review provide an adequate but preliminary evidence base for informing policy and practice. There is a need for more primary studies with stronger designs, more reliable outcome measures and more sophisticated analytic techniques.

### 4.2 Strengths and limitations

The major strength of this scoping review was the breadth of our literature search. We searched 11 data bases for all types of research studies published between 2006–January 2020. This yielded studies covering all regions of Brazil, with study samples showing diverse characteristics. However, our scoping review was limited to published studies. Our study was also limited by the quality and characteristics of the included studies. For example, the use of different time frames for measuring mammography uptake and the use of widely varying sets of predictors in the regression models makes meaningful summaries and comparisons between studies difficult if not impossible. For example, 24 of the 41 factors that were investigated were included in three or fewer studies. Moreover, only 11 of the 22 studies measured mammography screening within the last 2 years, thus limiting our ability to focus on factors that predict mammography screening according to national guidelines. These limitations should be kept in mind when interpreting our results.

### 4.3 Implications for nursing and health policy

Even though mammography screening is a publicly funded healthcare service in Brazil, our results suggest that there may still be barriers related to socio‐economic status, such as a lack of transportation or the opportunity to leave work for a medical appointment. Public health services should consider strategies to make mammography screening more accessible, such as a more convenient location and scheduling of mammography clinics. Diverse messaging may also be useful in reaching various subpopulations. However, the wide range in mammography adherence rates across regions of the country also raises larger policy questions about structural factors and differential access to mammography screening.

## 5 CONCLUSION

This review synthesized the literature on factors related to mammography adherence among women in Brazil. We identified several predictors of adherence/non‐adherence: age, urban/rural household location, region of the country, income, health insurance and having a recent medical appointment. Our results reinforce the findings of studies in other countries regarding the importance of socio‐economic factors at the individual level for mammography uptake (Akinyemiju, 2012), but also suggest a need to examine structural factors that may have an impact on access to screening. Moving forward, it will also be important to move beyond prediction to understanding, for example, using structural equation modelling and qualitative research methods.

## CONFLICT OF INTEREST

No conflict of interest has been declared by the authors.

## AUTHORS CONTRIBUTIONS

Study design: CBM, VSD, AFH, AFCF. Data collection: CBM. Data analysis: CBM, VSD, AFH. Study supervision: VSD, AFH, JS. Manuscript writing: CBM, VSD, AFH. Critical revisions for important intellectual content: VSD, AFH.

## ETHICAL APPROVAL

This scoping review drew on published studies only, and did not involve human participants; therefore, it did not require ethical review.

## Supporting information

File S1

## Data Availability

All data generated or analysed during this study are included in this published article (and its supplementary information File S1).
