# Supplementary material for: Factors related to mammography adherence among women in Brazil: A scoping review
Source: Nurs Open. 2020 Nov 24;8(5):2035–49. doi: 10.1002/nop2.706 (PMC8363398; doi:10.1002/nop2.706)
Supplement: Supplementary file 1 — File S1 [file NOP2-8-2035-s001.docx]

**Supplemental File 1.**

**Search Strategy for** “F**actors related to mammography adherence among women in Brazil: a scoping review”**

**MEDLINE (through Ovid) Search:**

Database: Ovid MEDLINE(R) Epub Ahead of Print, In-Process & Other Non-Indexed Citations, Ovid MEDLINE(R) Daily and Ovid MEDLINE(R) <1946 to Present>

Search Strategy:

--------------------------------------------------------------------------------

#1 mammogra*.mp.

#2 (Brazil).mp. [mp=title, abstract, original title, name of substance word, subject heading word, keyword heading word, protocol supplementary concept word, rare disease supplementary concept word, unique identifier, synonyms]

#3 #1 AND #2

#4 Limit #3 by year 2006-2019

**PubMed Search:**

Recent queries in pubmed

Search, Query, Items found, Time

Search Strategy:

--------------------------------------------------------------------------------

#1 "mammography" [All Fields] OR “mammogram”

#2 "Brazil"[All Fields]

#3 #1 AND #2

#4 Limit #3 by year 2006-2019

**Web of Science Core Collection Search:**

Indexes: SCI-EXPANDED, SSCI, A&HCI, CPCI-S, CPCI-SSH, ESCI Timespan: All years.

Search Strategy:

--------------------------------------------------------------------------------

#1 mammogra*[TOPIC]

#2 Brazil*[ TOPIC]

#3 #1 AND #2

#4 Limit #3 by year 2006-2019

**CINAHL** - **Cumulative Index to Nursing and Allied Health Literature (through EBSCOhost) Search:**

Interface - EBSCOhost Research Databases

Search Screen - Advanced Search

Database - CINAHL Complete

Search Strategy:

--------------------------------------------------------------------------------

#1 mammogra*[tx]

#2 Brazil*[tx]

#3 #1 AND #2

#4 Limit #3 by year 2006-2019

**Elsevier Science Direct Search:**

Search Strategy:

--------------------------------------------------------------------------------

#1 mammogra*[All Fields]

#2 Brazil*[All Fields]

#3 #1 AND #2

#4 Limit #3 by year 2006-2019

**LILACS (through BVS) Search:**

BIREME/OPAS/OMS - Biblioteca Virtual em Saúde. Database: LILACS

Search Strategy:

--------------------------------------------------------------------------------

#1 mammography [keywords]

#2 mammogram [keywords]

#3 Brazil [keywords]

#4 #1 OR #2

#5 #4 AND #3

#6 Limit #5 by year 2006-2019

**Scielo Search:**

Search Strategy:

--------------------------------------------------------------------------------

#1 mammogra* [All indexes]

#2 Brazil [All indexes]

#3 #1 AND #2

#4 Limit #3 by year 2006-2019

**Cancerlit Search:**

Search Strategy:

--------------------------------------------------------------------------------

#1 “mammography” [keywords]

#2 “Brazil” [keywords]

#3 #1 AND #2

#4 Limit #3 by year 2006-2019

**BDEnf (through BVS) Search:**

BIREME/OPAS/OMS - Biblioteca Virtual em Saúde. Database: BDENF

Search Strategy:

--------------------------------------------------------------------------------

#1 mammography [keywords]

#2 mammogram [keywords]

#3 Brazil [keywords]

#4 #1 OR #2

#5 #4 AND #3

#6 Limit #5 by year 2006-2019

**Med Carib (through BVS) Search:**

BIREME/OPAS/OMS - Biblioteca Virtual em Saúde. Database: MedCarib

Search Strategy:

--------------------------------------------------------------------------------

#1 mammography [keywords]

#2 mammogram [keywords]

#3 Brazil [keywords]

#4 #1 OR #2

#5 #4 AND #3

#6 Limit #5 by year 2006-2019

**PAHO (through BVS) Search:**

BIREME/OPAS/OMS - Biblioteca Virtual em Saúde. Database: PAHO

Search Strategy:

--------------------------------------------------------------------------------

#1 mammography [keywords]

#2 mammogram [keywords]

#3 Brazil [keywords]

#4 #1 OR #2

#5 #4 AND #3

#6 Limit #5 by year 2006-2019
